# Identification of Core Gene Expression Signature and Key Pathways in Colorectal Cancer

**DOI:** 10.3389/fgene.2020.00045

**Published:** 2020-02-21

**Authors:** Xiang Ding, Houyu Duan, Hesheng Luo

**Affiliations:** Department of Gastroenterology, Renmin Hospital, Wuhan University, Wuhan, China

**Keywords:** MAD2L1, colorectal cancer, bioinformatics analysis, proliferation, cell cycle, apoptosis

## Abstract

**Objective:**

Colorectal cancer (CRC) is considered the most prevalent malignant tumor that contributes to high cancer-related mortality. However, the signaling pathways involved in CRC and CRC-driven genes are largely unknown. We sought to discover a novel biomarker in CRC.

**Materials and Methods:**

All clinical CRC samples (n = 20) were from Renmin Hospital of Wuhan University. We first selected MAD2L1 by integrated bioinformatics analysis of a GSE dataset. Next, the expression of MAD2L1 in tissues and cell lines was verified by quantitative real-time PCR. The effects of MAD2L1 on cell growth, proliferation, the cell cycle, and apoptosis were examined by *in vitro* assays.

**Results:**

We identified 683 shared DEGs (420 upregulated and 263 downregulated), and the top twenty genes (CDK1, CCNA2, TOP2A, PLK1, MAD2L1, AURKA, BUB1B, UBE2C, TPX2, RRM2, KIF11, NCAPG, MELK, NUSAP1, MCM4, RFC4, PTTG1, CHEK1, CEP55, DTL) were selected by integrated analysis. These hub genes were significantly overexpressed in CRC samples and were positively correlated. Our data revealed that the expression of MAD2L1 in CRC tissues is higher than that in normal tissues. MAD2L1 knockdown significantly suppressed CRC cell growth by impairing cell cycle progression and inducing cell apoptosis.

**Conclusion:**

MAD2L1, as a novel oncogenic gene, plays a role in regulating cancer cell growth and apoptosis and could be used as a new biomarker for diagnosis and therapy in CRC.

## Introduction

Colorectal cancer (CRC) is currently a major public health problem in medicine today. CRC is one of the most frequently occurring malignancies worldwide, with more than 777,000 new cases expected in 2015 and almost 350,000 deaths in developed countries (Ferlay et al., 2015). The risk of developing colorectal cancer depends on different variables that can be classified into lifestyle or behavioral factors and genetically determinant factors. Similar to other cancers, CRC is considered a polyphase disease in which gene distortions, cellular contexts, and environmental influences concur with tumor initiation, progression, and metastasis (Aran et al., 2016). Increasing evidence shows that multiple genes and cellular pathways are involved in the occurrence and development of CRC. Until now, a lack of knowledge about the exact molecular mechanisms underlying CRC progression has limited the ability to treat advanced disease. On the other hand, so far, the main clinical screening methods for CRC involve endoscopic screening, especially colonoscopy. Colonoscopy has shortcomings such as poor patient compliance, the influence of family history, inconvenience, and high cost and risk. Therefore, it is of great significance to understand the molecular mechanisms of CRC proliferation, apoptosis and invasion in order to develop more effective diagnostic and therapeutic strategies.

The recently adopted high-throughput gene microarray analysis of tumors and samples from patients and healthy people allows us to share and explore global molecular tumors at different levels of the landscape from somatic mutations and copy number changes to genome-level gene expression at the transcriptome level, as well as epigenetic changes (Liu et al., 2017; Sun et al., 2017; Chen et al., 2017). In this study, we downloaded the GSE117606 dataset from the Gene Expression Omnibus (GEO, http://www.ncbi.nlm.nih.gov/geo) database using R software for the comprehensive identification of differentially expressed genes (DEGs). Then, we established a protein–protein interaction (PPI) network of DEGs to screen out the first 20 hub genes with a high degree of connectivity. In addition, we also analyzed Gene Ontology involving the biological processes (BPs), molecular functions (MFs), and cellular components (CCs) of the DEGs as well as their KEGG pathways. The potential correlation and expression levels were analyzed *via* Gene Expression Profiling Interactive Analysis (GEPIA) (http://gepia.cancer-pku.cn/index.html).

Our data showed that the expression of MAD2L1 is significantly higher in CRC tissues than in normal tissues. The cell cycle progression could be slowed, and apoptosis could be induced by knocking down MAD2L1, which directly leads to the inhibition of the growth of CRC cells. In conclusion, MAD2L1 can be used as a new diagnostic indicator and guide the combined treatment of CRC.

## Materials and Methods

### Microarray Data

We downloaded the gene expression profile of GSE117606 from the GEO database, a free public database. The GSE117606 dataset has a total of 208 samples, containing 74 CRC samples and 65 normal colon tissues and was based on the Agilent GPL25373 platform (HT_HG-U133_Plus_PM) Affymetrix HT HG-U133+ PM Array Plate (CDF: HTHGU133PlusPM_Hs_ENTREZG_20) by Joke Reumers et al. We also downloaded the Series Matrix File of GSE117606 from the GEO database.

### Data Preprocessing

The expression values of all probes in each sample were reduced to a single value by determining the mean expression value *via* the aggregate function method (Li, 1991). Missing data were assigned using the k-nearest neighbor method (Altman, 1992). Quantile normalization for complete data was performed using the preprocessCore package in Bioconductor (Bolstad et al., 2003). When many probes were mapped to a gene, the median of the data was defined as the level of expression of that gene. However, when many genes were located by a probe, the probe was considered to lack specificity and was removed from the analysis.

### Identification of DEGs

We utilized the “limma” R package (Ritchie et al., 2015) to identify the DEGs between CRC samples and normal ovarian samples. Adjusted *P <* 0.05 and |log fold change (FC)| > 1 were chosen as the cutoff criteria. The adjusted *P*-value (adj. *P*) was applied to help correct false positives. The heat map and volcano plot were drawn with the “gplots” package in R 3.5.3 (Galili et al., 2018).

A total of 683 DEGs were found, including 420 upregulated genes and 263 downregulated genes, and we selected the top 20 genes with a high degree of connectivity as hub genes.

### Gene Ontology and KEGG Pathway Analysis of DEGs

Gene Ontology (GO) analysis can be used to annotate genes and their products with cellular components (CCs), molecular functions (MFs), biological pathways (BPs), and other functions (Gaudet et al., 2017). The Kyoto Encyclopedia of Genes and Genomes (KEGG) is a collection of databases that address genomic and biological pathways related to diseases and drugs. KEGG is essentially a resource for the comprehensive understanding of biological systems and some high-level genomic functional information (Kanehisa, 2002). Database for Annotation, Visualization, and Integrated Discovery (DAVID, http://david.ncifcrf.gov) (version 6.8) is an online biological information database that integrates a large amount of biological data and related analysis tools, providing systematic and comprehensive biological function annotation information for high-throughput gene expression (Huang et al., 2007). P < 0.05 was used as the cut-off criterion for statistically significant differences. To visualize the key molecular functions, biological processes, cellular components, and KEGG pathways of the DEGs, the DAVID online database was used to perform biological analysis.

### PPI Network and Module Analysis

The Search Tool for the Retrieval of Interacting Genes/Proteins (STRING) is an online tool that was designed to evaluate and integrate protein–protein interaction (PPI) information, such as physical and functional associations. To date, a total of 9,643,763 proteins from 2,031 organisms have been covered in STRING version 11.0 (Szklarczyk et al., 2015). To evaluate the interrelationships among these DEGs, we first drew the network of DEGs in STRING and then visualized the PPI network by using Cytoscape software. Moreover, we set the maximum number of interacting bodies to 0 and used a confidence score of 0.7 as the cut-off criterion. Additionally, the Molecular Complex Detection (MCODE) app was also employed to select modules of the PPI network in Cytoscape according to node score cut-off = 0–2, degree cut-off = 2, max.depth = 100, and k−core = 2. With DAVID, the gene pathways of the three modules were analyzed. Additionally, 20 hub genes were mapped into STRING according to a confidence score ≥0.4 and a maximum number of interactors ≤5. We also used GO and KEGG pathway analysis to investigate their underlying information.

### Comparison of the Hub Genes’ Expression Levels

GEPIA (http://gepia.cancer-pku.cn/index.html) is a newly developed interactive web server designed by Zefang Tang, Chenwei Li, and Boxi Kang of the Zhang Lab, Peking University, designed to analyze the RNA sequence expression data of 9,736 tumors and 8,587 normal samples from the TCGA and GTEx projects using a standard processing pipeline. GEPIA provides customizable capabilities, such as tumor/normal differential expression analysis, profiling by cancer type or pathological stages, patient survival analysis, similar gene testing, correlation analysis, and dimensional reduction analysis (Tang et al., 2017). In our study, we mainly used boxplots to visualize hub gene expression in CRC and normal colon tissues. Then, we analyzed the top 20 hub genes' correlation with a scatter plot. The Human Protein Atlas (HPA, https://www.proteinatlas.org/) is a Swedish-based program initiated in 2003 with the aim of mapping all human proteins in cells, tissues, and organs using the integration of various omics technologies, including antibody-based imaging, mass spectrometry-based proteomics, transcriptomics, and systems biology (Uhlen et al., 2017). We further verified the expression of MAD2L1 by obtaining immunohistochemical data based on the HPA in patients with or without CRC.

### Gene Set Enrichment Analysis

Gene Set Enrichment Analysis (GSEA) is a computational method for exploring whether a given gene set is significantly enriched in a group of gene markers ranked by their relevance with a phenotype of interest. The curated KEGG pathway V5.2 data set was used to compare the impaired pathways in normal and colon cancer samples. In addition, the gene sets with fewer than 15 genes or more than 500 genes were excluded. The phenotype label was set as colon cancer *versus* control. The t-statistic mean of the genes was computed in each KEGG pathway using a permutation test with 1,000 replications. The upregulated pathways were defined by a normalized enrichment score (NES) > 0, and the downregulated pathways were defined by an NES <0. Pathways with an FDR P value ≤1 were considered significantly enriched.

### Validation Based on CRC Clinical Samples

To further verify the data from GEO, we conducted quantitative real-time PCR (qRT-PCR) to quantify the expression level of MAD2L1 in clinical CRC patient samples (n = 20) from Renmin Hospital of Wuhan University (Wuhan, China). Written informed consent was obtained from all patients. This study was approved by the Institute Research Ethics Committee of Renmin Hospital of Wuhan University.

### Cell Lines and Cell Transfection

All cell lines, including the normal cell line NCM460 and the CRC cell lines HT-29, HCT116, SW620, and SW480, were purchased from Bioyear Biotechnology. The cells were cultured in RPMI-1640 medium supplemented with 10% FBS (Thermo Fisher Scientific). All cells were maintained in a humidified incubator with 5% CO_2_ at 37°C. A total of 1 × 10^4^ cells/ml were plated approximately 24 h before transfection. Once the cells reached 40%–60% confluence in each well of a 96-well plate, the cells were transfected with 2.5 nM siRNA/NC (RiboBio, Guangzhou, China) using Lipofectamine 2000 (Thermo Fisher Scientific) at the indicated concentrations according to the manufacturer's instructions. Six hours later, the culture medium was replaced with fresh medium containing 10% FBS. The cells were harvested after 24 h of transfection for the following assays.

The siRNA sequences were as follows:

Si-h-MAD2L1: forward, 5'-GGGUCCAAAGUUGAGUGAGUCUUGAdTdT-3'; reverse, 5'-CGGACUCACCUUGCUUGUAACUACUdTdT-3'.

### RNA Extraction, Reverse Transcription (RT)-PCR, and qRT-PCR

Total RNA was extracted from cells using TRIzol reagent (Invitrogen™). Reverse-transcribed complementary DNA was synthesized using the PrimeScript™RT Reagent Kit (Takara). The RT-PCR conditions were 37°C for 15 min, 85°C for 5 s, and held at 4°C. After the dilution (1:4) of cDNA with nuclease-free water, qRT-PCR was performed by a StepOne™ Real-Time PCR system and SYBR^®^ Premix Ex Taq™. The mixes were predenatured at 95°C for 1 min, followed by 40 cycles of denaturation at 95°C for 15 s and 72°C for 45 s. The results were normalized to GAPDH expression. The relative expression level of MAD2L1 was calculated by the 2^−ΔΔCt^ method.

The primers used for qRT-PCR were as follows: GAPDH forward, 5'-CATCATCCCTGCCTCTACTGG-3'; and reverse, 5'-GTGGGTGTCGCTGTTGAAGTC-3'; MAD2L1 forward, 5'-GCAAAAGATGACAGTGCACCC-3'; and reverse, 5'-GTGGTCCCGACTCTTCCCAT -3'.

### Colony Formation Assay

Twenty-four hours after SW620 cells were infected with siRNA, approximately 300 cells were seeded on each well of a six-well plate. The cells were allowed to incubate at 37°C for 14 days. Then, the cells were fixed, stained with crystal violet, and photographed. ImageJ software (1.48 u; National Institutes of Health) was used to count the number of clones per well.

### Cell Cycle Analysis

Twenty-four hours after siRNA interference, SW620 cells were harvested, centrifuged, and resuspended in 1× PBS. The cells were fixed in 70% ethanol overnight. On the second day, after being washed with 1× PBS solution and centrifuged, the cells were resuspended in 1× PBS solution and incubated with RNaseA at 37°C for 30 min. Finally, the cells were stained with propidium iodide and analyzed by a FACSCalibur system (BD Biosciences).

### Apoptosis Analysis

SW620 cells were transfected with siRNA for 24 h, harvested, and centrifuged. Then, the supernatant was removed and resuspended in 1× PBS solution. This procedure was repeated three times with 1 × 10^6^ cells per well, and then the cells were stained with an Annexin V/FITC and PI kit. After staining, the cells were analyzed with a FACSCalibur system (BD Biosciences).

### Statistical Analysis

All experiments were performed at least three times, and each independent test was carried out in triplicate for each condition under the protocol and according to the manufacturer's instructions. All statistical analyses were performed using PASW Statistics 19.0 (IBM) or GraphPad Prism 6 software (GraphPad Software, Inc.).

## Results

### Identification of DEGs and Hub Genes

**Figure 1:**
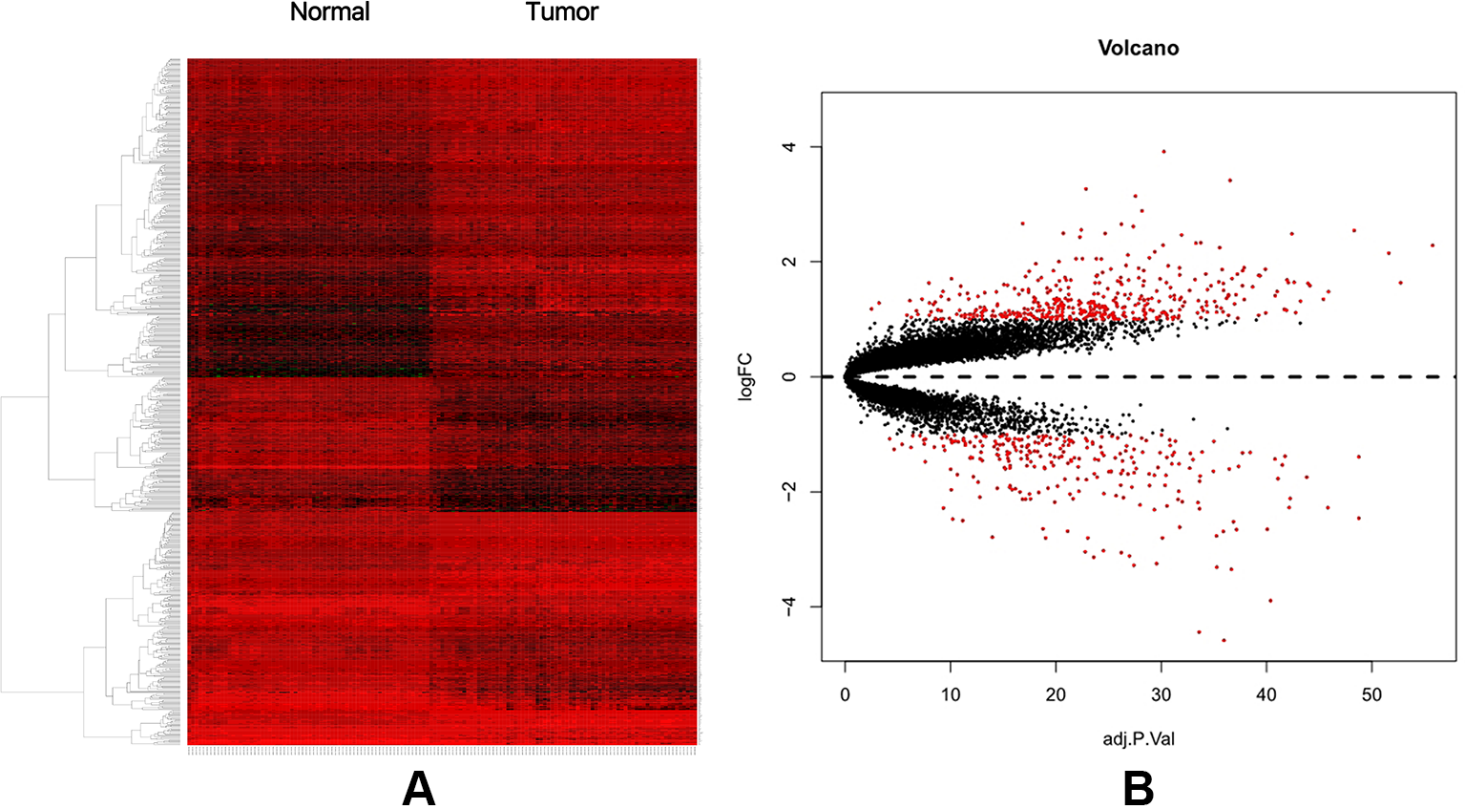
**(A)** Heat map of DEGs. **(B)** Volcano plot of genes detected in CRC. Red means upregulated and downregulated DEGs; black means no difference.

A total of 74 CRC samples and 65 normal samples were analyzed. The series from each chip was analyzed separately using R software, and finally, the DEGs, using adjusted P value < 0.05 and logFC ≥ 1 or logFC ≤ −1 as the cut-off criteria, were identified. A total of 683 DEGs were identified after analyzing GSE117606, 420 of which were upregulated genes, and 263 were downregulated (**Figure 1B**). **Figure 1A** shows the performance level of the DEGs with a fold change of 1. In addition, 20 hub genes were identified from high to low according to their degree of connectivity (**Table 1**).

**Table 1:** Top 20 hub genes with higher degree of connectivity.

Gene	Degree of connectivity	Adjusted p value
CDK1	55	4.64E-50
CCNA2	46	8.09E-28
TOP2A	41	3.81E-25
PLK1	40	3.35E-22
MAD2L1	39	2.61E-21
AURKA	38	1.39E-30
BUB1B	37	3.23E-38
UBE2C	37	2.11E-28
TPX2	36	1.57E-33
RRM2	36	2.23E-22
KIF11	35	4.54E-31
NCAPG	34	2.35E-27
MELK	34	1.01E-25
NUSAP1	33	3.24E-28
MCM4	29	2.76E-26
RFC4	29	3.04E-22
PTTG1	29	1.78E-24
CHEK1	29	2.05E-37
CEP55	29	1.66E-24
DTL	28	5.48E-25

### GO Function and KEGG Pathway Enrichment Analysis

To obtain a more comprehensive and in-depth understanding of the selected DEGs, we analyzed the GO function and KEGG pathway enrichment by DAVID. After importing all DEGs into DAVID, we discovered the functions of the upregulated DEGs and downregulated DEGs by GO analysis. More specifically, these DEGs were mainly enriched in biological processes (BPs) involving collagen catabolic process, extracellular matrix organization, collagen fibril organization, cell division, and G1/S transition of the mitotic cell cycle for the upregulated genes; and bicarbonate transport, muscle contraction, regulation of intracellular pH, chloride transmembrane transport, and one-carbon metabolic process for the downregulated genes. Regarding function (MF), the DEGs were involved in extracellular matrix structural constituent, extracellular matrix binding, platelet-derived growth factor binding, chemokine activity, and calcium ion binding for the upregulated genes; and chloride channel activity, carbonate dehydratase activity, NAD binding, hormone activity, and intracellular calcium activated chloride channel activity for the downregulated genes. In addition, GO cell component (CC) analysis revealed that the upregulated DEGs were principally enriched in the proteinaceous extracellular matrix, extracellular region, extracellular space, collagen trimer, and extracellular matrix, while the downregulated DEGs were mainly enriched in extracellular exosomes, extracellular space, integral components of the plasma membrane, brush border membrane, and apical plasma membrane (**Table 2**).

**Table 2:** Gene Ontology analysis of differentially expressed genes associated with colorectal cancer.

Expression	Category	Term	Count	%	*P* value	FRD
Upregulated	GOTERM_BP_DIRECT	GO:0030574~collagen catabolic process	19	3.38	6.88E-16	1.14E-12
	GOTERM_BP_DIRECT	GO:0030198~extracellular matrix organization	25	4.45	4.39E-12	7.53E-09
	GOTERM_BP_DIRECT	GO:0030199~collagen fibril organization	13	2.31	1.60E-11	2.75E-08
	GOTERM_BP_DIRECT	GO:0051301~cell division	26	4.63	1.28E-07	2.19E-04
	GOTERM_BP_DIRECT	GO:0000082~G1/S transition of mitotic cell cycle	14	2.49	2.40E-07	4.11E-04
	GOTERM_CC_DIRECT	GO:0005201~extracellular matrix structural constituent	12	2.14	1.18E-07	1.73E-04
	GOTERM_CC_DIRECT	GO:0050840~extracellular matrix binding	8	1.42	7.27E-07	1.06E-03
	GOTERM_CC_DIRECT	GO:0048407~platelet-derived growth factor binding	6	1.07	1.55E-06	2.26 E-03
	GOTERM_CC_DIRECT	GO:0008009~chemokine activity	9	1.60	6.77E-06	9.87 E-03
	GOTERM_CC_DIRECT	GO:0005509~calcium ion binding	31	5.52	2.18E-04	0.32
	GOTERM_MF_DIRECT	GO:0005578~proteinaceous extracellular matrix	28	4.98	4.16E-12	5.63E-09
	GOTERM_MF_DIRECT	GO:0005576~extracellular region	71	12.63	2.09E-10	2.83E-07
	GOTERM_MF_DIRECT	GO:0005615~extracellular space	61	10.85	2.12E-09	2.87E-06
	GOTERM_MF_DIRECT	GO:0005581~collagen trimer	14	2.49	3.07E-08	4.16E-05
	GOTERM_MF_DIRECT	GO:0031012~extracellular matrix	22	3.91	4.80E-07	6.49E-04
Downregulated	GOTERM_BP_DIRECT	GO:0015701~bicarbonate transport	8	2.60	1.19E-06	1.91 E-03
	GOTERM_BP_DIRECT	GO:0006936~muscle contraction	10	3.25	8.46E-06	0.01
	GOTERM_BP_DIRECT	GO:0051453~regulation of intracellular pH	6	1.95	8.39E-05	0.13
	GOTERM_BP_DIRECT	GO:1902476~chloride transmembrane transport	8	2.60	1.75E-04	0.28
	GOTERM_BP_DIRECT	GO:0006730~one-carbon metabolic process	5	1.63	5.23E-04	0.83
	GOTERM_CC_DIRECT	GO:0005254~chloride channel activity	6	1.95	4.91E-04	6.73
	GOTERM_CC_DIRECT	GO:0004089~carbonate dehydratase activity	4	1.30	5.82E-04	7.98
	GOTERM_CC_DIRECT	GO:0051287~NAD binding	5	1.63	1.13 E-03	1.54
	GOTERM_CC_DIRECT	GO:0005179~hormone activity	6	1.95	5.77 E-03	7.65
	GOTERM_CC_DIRECT	GO:0005229~intracellular calcium activated chloride channel activity	3	9.75	0.02	19.61
	GOTERM_MF_DIRECT	GO:0070062~extracellular exosome	69	2.24	1.68E-08	2.13E-05
	GOTERM_MF_DIRECT	GO:0005615~extracellular space	38	12.35	3.90E-06	4.95 E-03
	GOTERM_MF_DIRECT	GO:0005887~integral component of plasma membrane	39	12.68	4.80E-06	6.09 E-03
	GOTERM_MF_DIRECT	GO:0031526~brush border membrane	7	2.28	3.85E-05	0.05
	GOTERM_MF_DIRECT	GO:0016324~apical plasma membrane	13	4.23	2.91E-04	0.37

**Figure 2:**
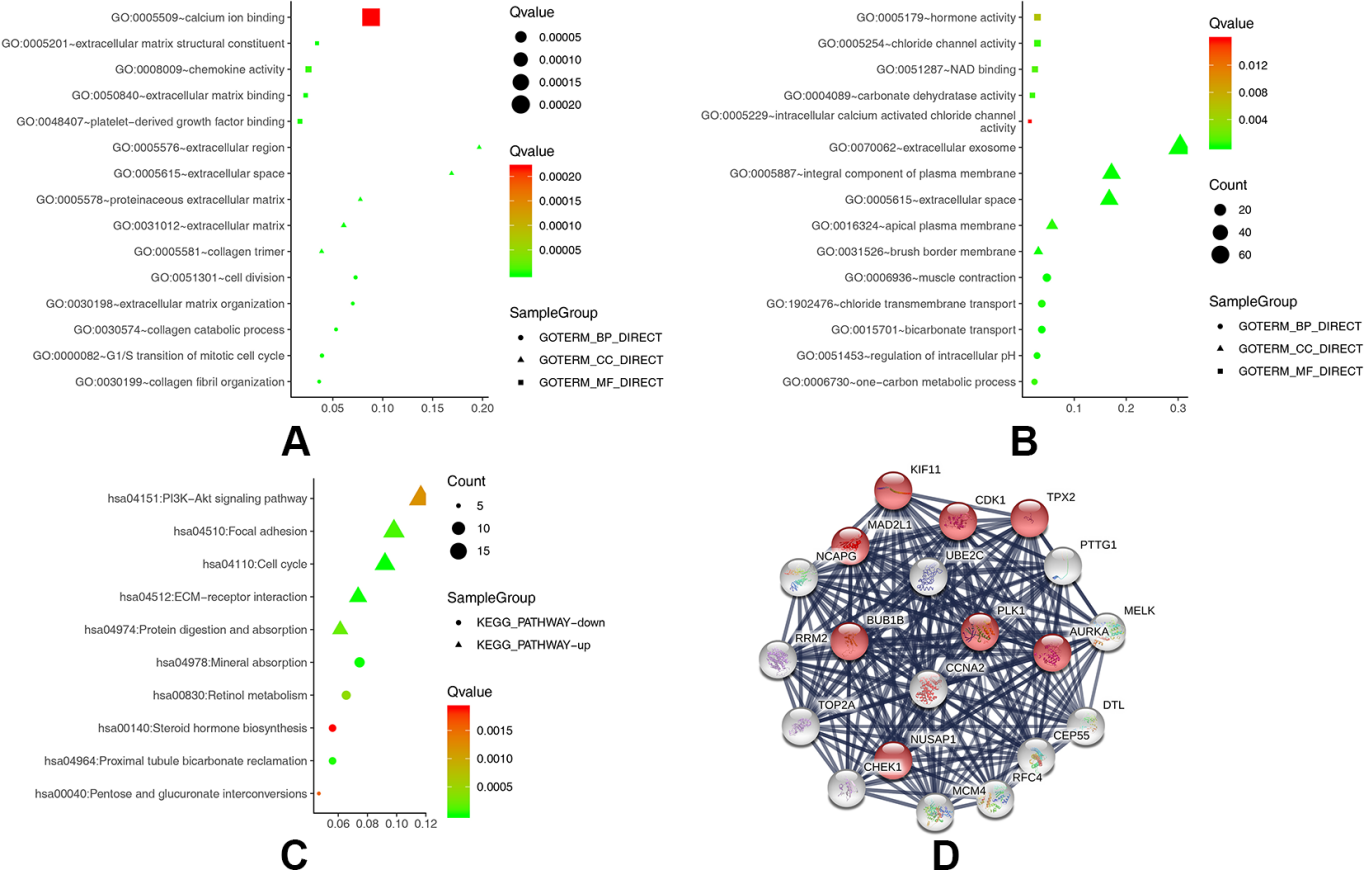
**(A)** GO analysis of upregulated DEGs. **(B)** GO analysis of downregulated DEGs. **(C)** KEGG pathway of DEGs. **(D)** The protein–protein interaction (PPI) network of the top 20 hub genes.

**Table 3** shows the most significantly enriched KEGG pathways of the upregulated and downregulated DEGs. The upregulated DEGs were enriched in the cell cycle, ECM-receptor interaction, focal adhesion, protein digestion and absorption, and the PI3K-Akt signaling pathway, while the downregulated DEGs were enriched in mineral absorption, proximal tubule bicarbonate reclamation, retinol metabolism, pentose and glucuronate interconversions, and steroid hormone biosynthesis. **Figures 2A–C** present a GO and KEGG pathway enrichment plot of CRC.

**Table 3:** KEGG pathway analysis of differentially expressed genes associated with colorectal cancer.

Category	Term	Count	%	P value	Genes	FRD
Upregulated	hsa04110: Cell cycle	15	0.03	1.06E-06	CDK1, DBF4, SKP2, CHEK1, PTTG1, MCM4, WEE1, YWHAG, CCND1, MAD2L1, MCM7, PLK1, PCNA, BUB1B, CCNA2	0.00133
	hsa04512: ECM-receptor interaction	12	0.02	5.25E-06	COL4A1, ITGAV, COMP, COL3A1, COL1A2, ITGA2, COL1A1, COL11A1, THBS2, COL5A2, COL5A1, SPP1	0.00658
	hsa04510: Focal adhesion	16	0.03	9.37E-05	COL4A1, COL3A1, MET, ITGA2, COL5A2, COL5A1, CCND1, ITGAV, COMP, VEGFA, COL1A2, PDGFRB, COL1A1, THBS2, COL11A1, SPP1	0.11741
	hsa04974: Protein digestion and absorption	10	0.02	2.07E-04	COL4A1, COL7A1, COL3A1, COL1A2, COL12A1, COL1A1, COL11A1, COL5A2, COL5A1, COL10A1	0.25962
	hsa04151: PI3K-Akt signaling pathway	19	0.03	1.19 E-03	COL4A1, COL3A1, MET, ITGA2, COL5A2, COL5A1, DDIT4, YWHAG, EIF4EBP1, CCND1, ITGAV, COMP, VEGFA, COL1A2, PDGFRB, COL1A1, THBS2, COL11A1, SPP1	1.48049
Downregulated	hsa04978:Mineral absorption	8	0.03	4.05E-06	SLC26A3, TRPM6, CLCN2, MT1M, SLC9A3, MT1E, ATP1A2, MT1F	0.00484
	hsa04964:Proximal tubule bicarbonate reclamation	6	0.02	2.13E-05	SLC9A3, CA4, ATP1A2, CA2, SLC4A4, PCK1	0.02546
	hsa00830:Retinol metabolism	7	0.02	4.22E-04	ALDH1A1, UGT1A6, UGT2B17, ADH1C, DHRS9, ADH1B, UGT2B28	0.50270
	hsa00040:Pentose and glucuronate interconversions	5	0.02	1.55 E-03	UGT1A6, UGT2B17, AKR1B10, UGDH, UGT2B28	1.82991
	hsa00140:Steroid hormone biosynthesis	6	0.02	1.89 E-03	HSD3B2, UGT1A6, UGT2B17, HSD17B2, HSD11B2, UGT2B28	2.23810

KEGG, Kyoto Encyclopedia of Genes and Genomes; FDR, false discovery rate.

### Hub Genes and Module Screening of the PPI Network

**Figure 3:**
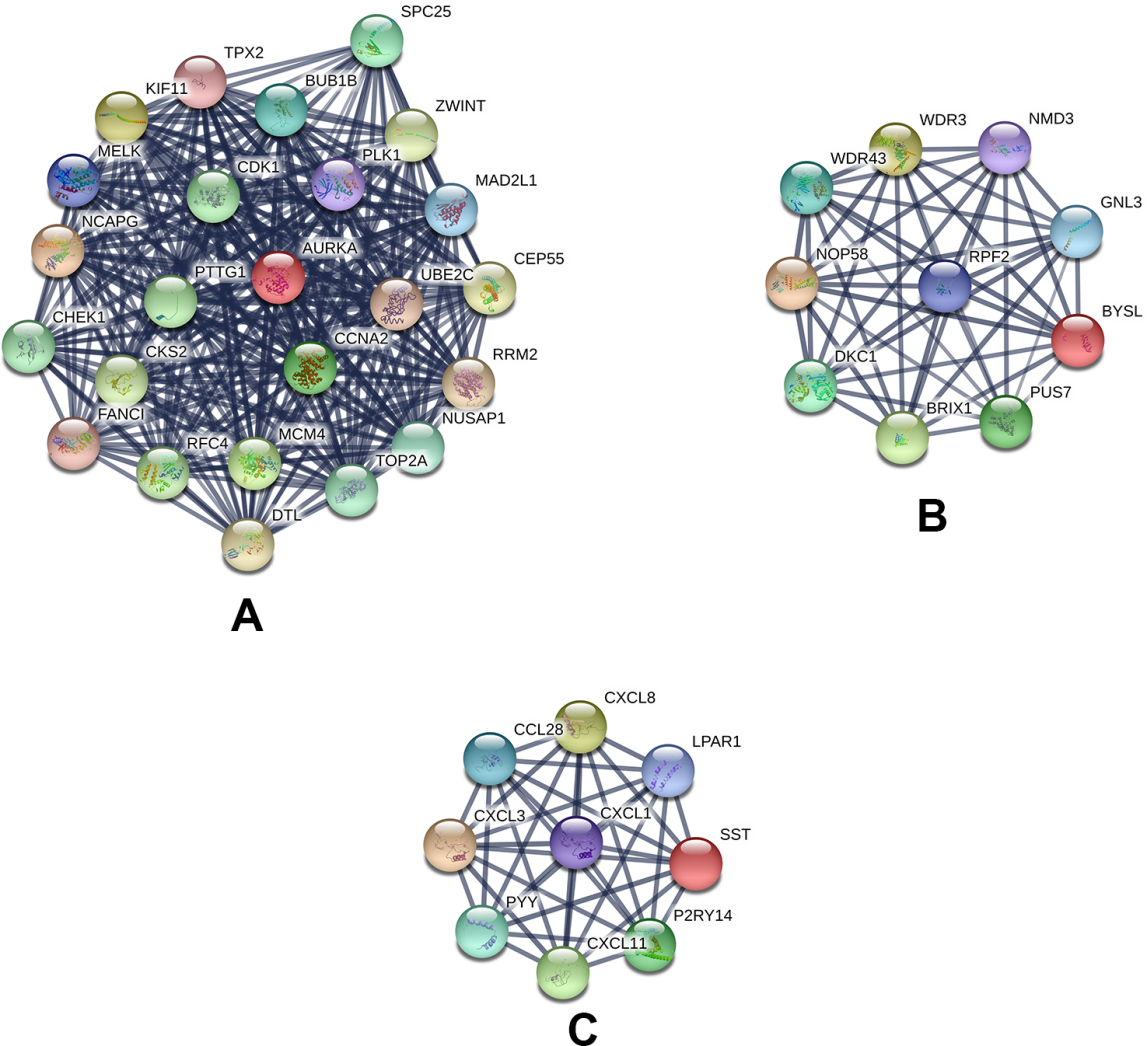
Top 3 modules from the protein–protein interaction network: **(A)** module 1, **(B)** module 2, **(C)** module 3.

Based on querying STRING protein information from the public database, we constructed a PPI network of the top 20 hub genes according to the degree of connectivity (**Figure 2D**). The top 20 hub genes with a high degree of connectivity were as follows: CDK1, CCNA2, TOP2A, PLK1, MAD2L1, AURKA, BUB1B, UBE2C, TPX2, RRM2, KIF11, NCAPG, MELK, NUSAP1, MCM4, RFC4, PTTG1, CHEK1, CEP55, and DTL. Based on the GO function and KEGG pathway analysis, we found that CDK1, MAD2L1, PLK1, BUB1B, CHEK1, PTTG1, CCNA2, and MCM4 were enriched in the cell cycle. To detect the most important module in this PPI network, we used the MCODE plug-in. The top 3 modules were selected (**Figure 3**). KEGG pathway analysis revealed that the top 3 modules were mainly associated with the cell cycle, ribosome biogenesis in eukaryotes, and the chemokine signaling pathway (**Table 4**).

**Table 4:** The enriched pathways of top 3 modules.

Category	Term	Count	%		P value	Genes	FRD
Module 1	hsa04110:Cell cycle	8		33.33	8.19E-10	CDK1, MAD2L1, PLK1, BUB1B, CHEK1, PTTG1, CCNA2, MCM4	6.49E-07
	hsa04114:Oocyte meiosis	5		20.83	4.10E-05	CDK1, MAD2L1, PLK1, AURKA, PTTG1	0.03253
	hsa04914:Progesterone-mediated oocyte maturation	4		16.67	5.10E-04	CDK1, MAD2L1, PLK1, CCNA2	0.40350
	hsa04115:p53 signaling pathway	3		12.5	6.80 E-03	CDK1, RRM2, CHEK1	5.26615
	hsa05166:HTLV-I infection	4		16.67	0.01	MAD2L1, BUB1B, CHEK1, PTTG1	8.25324
Module 2	hsa03008:Ribosome biogenesis in eukaryotes	6		60	2.88E-10	DKC1, WDR3, NOP58, WDR43, NMD3, GNL3	6.51E-08
Module 3	hsa04062:Chemokine signaling pathway	5		0.53	1.70E-05	CXCL1, CXCL3, CXCL8, CXCL11, CCL28	0.01435
	hsa04060:Cytokine-cytokine receptor interaction	5		0.53	4.89E-05	CXCL1, CXCL3, CXCL8, CXCL11, CCL28	0.04128
	hsa05134:Legionellosis	3		0.32	1.24 E-03	CXCL1, CXCL3, CXCL8	1.04102
	hsa05132:Salmonella infection	3		0.32	2.90 E-03	CXCL1, CXCL3, CXCL8	2.42586
	hsa04621:NOD-like receptor signaling pathway	2		0.21	5.56 E-02	CXCL1, CXCL8	38.32740

### The Expression Level and Correlation Analyses of the Twenty Hub Genes in GEPIA

**Figure 4:**
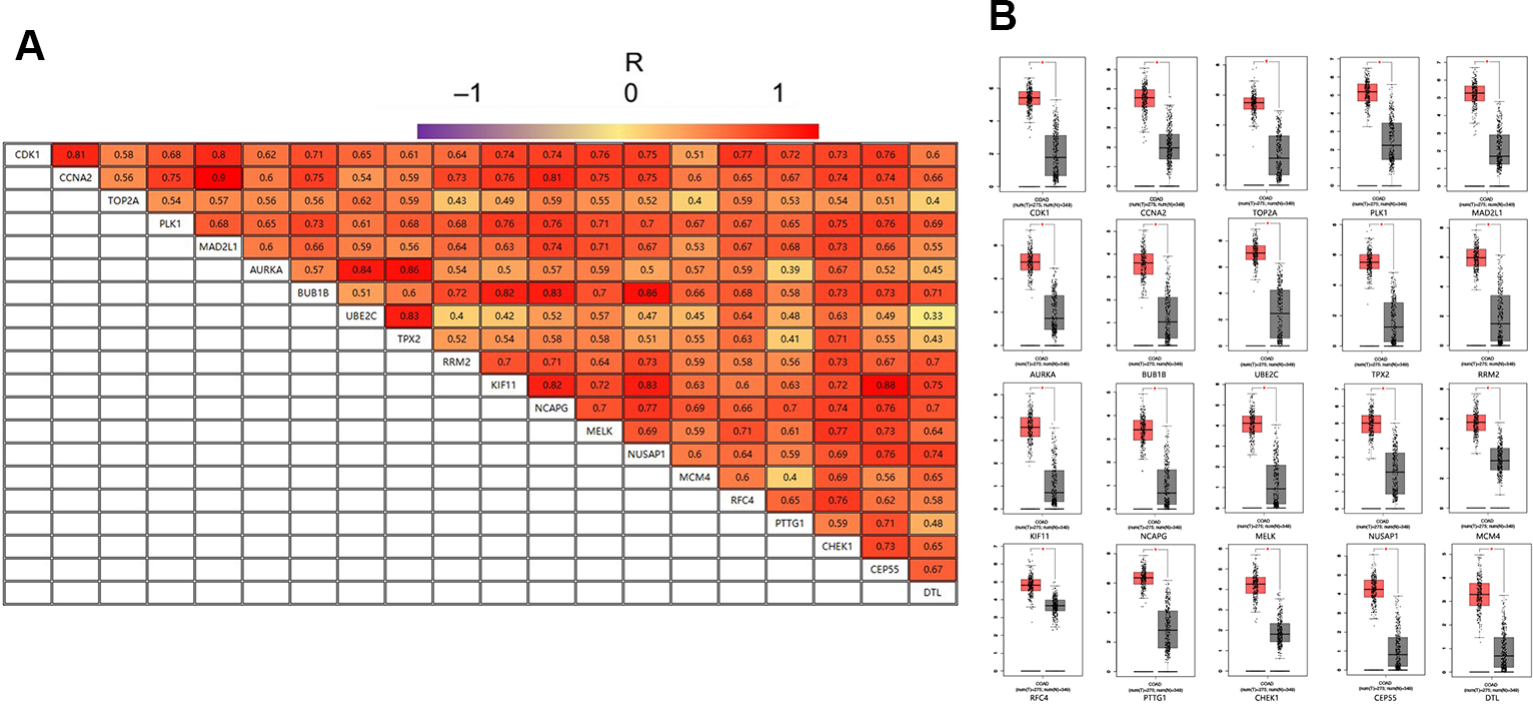
**(A)** The correlation analysis of the 20 hub genes. **(B)** Expression levels of the 20 hub genes in CRC compared to the normal samples. Notes: R is the Pearson correlation coefficient. Abbreviations: CRC, colorectal cancer.

GEPIA is an interactive online server for exploring large data sets from the TCGA and GTEx projects. To confirm the reliability of the twenty identified hub genes from the data sets, we used GEPIA to verify the correlation between them, and they were obviously positively correlated with each other in CRC (**Figure 4A**). GEPIA was also used to determine the expression levels of the top ten genes in CRC. **Figure 4B** shows that these genes were all significantly overexpressed in the colon cancer (COAD) samples compared to the normal samples.

### Gene Set Enrichment Analysis

**Figure 5:**
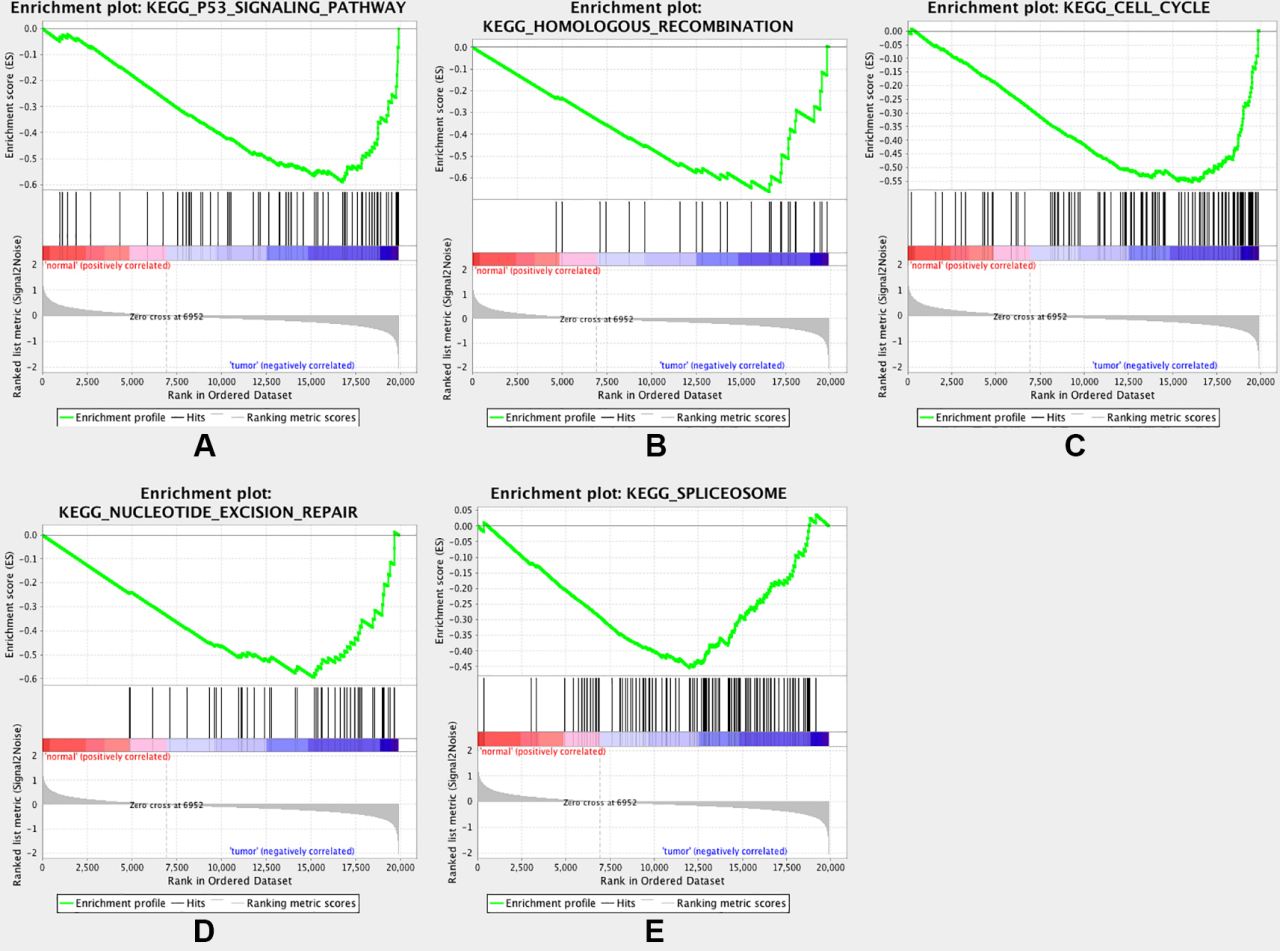
Gene set enrichment analysis (GSEA). Listed pictures are five representative functional gene sets enriched in CRC with MAD2L1 highly expressed.

To gain further insight into the functions of the DEGs, GSEA was conducted to map the DEGs into the KEGG pathway database. Under the cut-off criteria of FDR <0.05, |enrichment score (ES)| > 0.6, and gene size ≥100, the top five pathways were “p53 signaling pathway,” “homologous recombination,” “cell cycle,” “nucleotide excision repair”, and “spliceosome” (**Figure 5**).

### Expression Patterns of MAD2L1 in CRC

**Figure 6:**
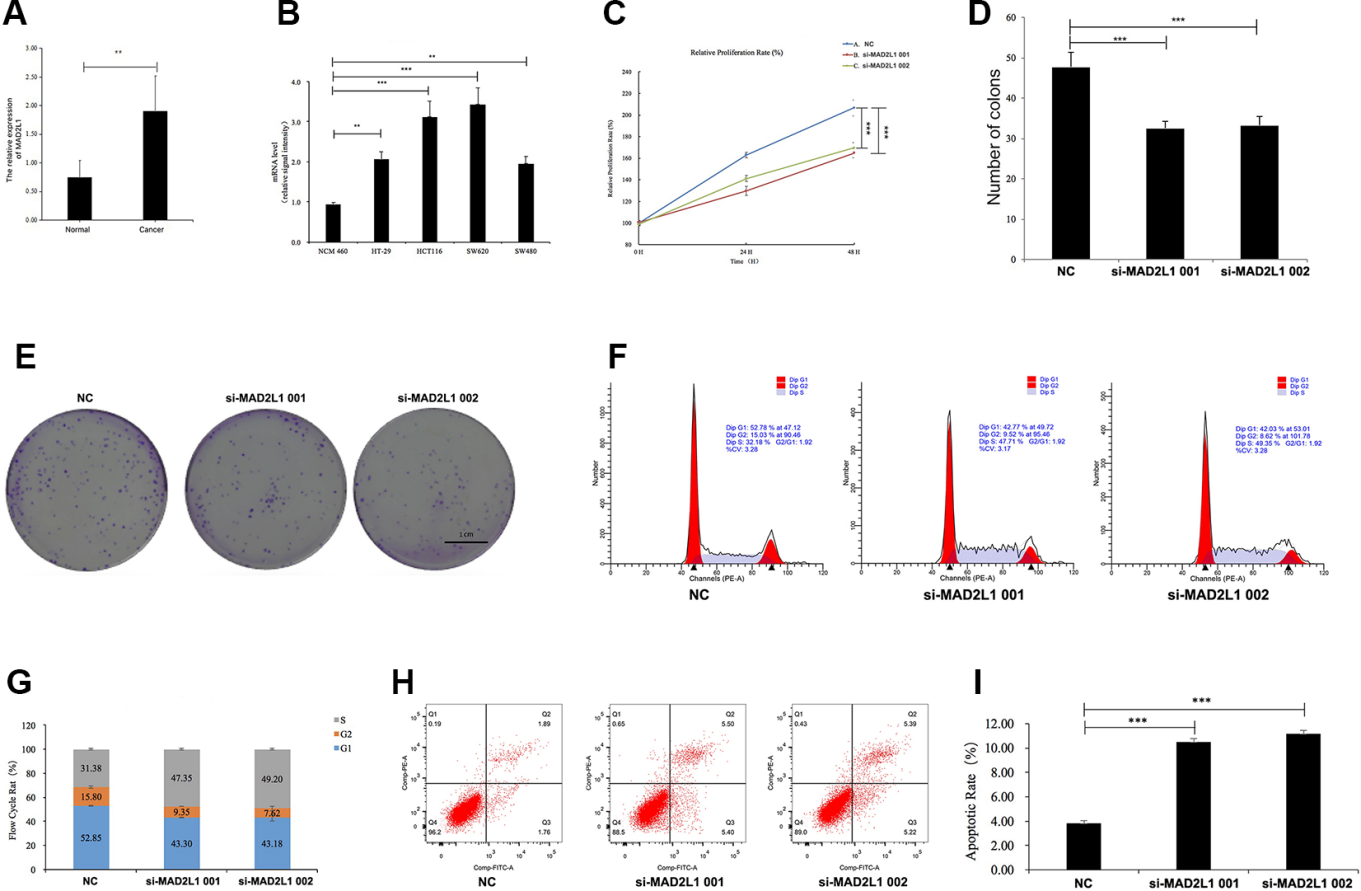
MAD2L1 knockdown suppressed colon cancer cell proliferation by impairing cell cycle progression and inducing apoptosis. Notes: **(A)** Expression level of MAD2L1 gene in 20 paired CRC tissues (n = 3; **P < 0.01; two-tailed t-test). **(B)** Expression level of MAD2L1 gene in colon normal cell line NCM460 and CRC cell line HT-29, HCT116, SW620 and SW480 (n = 3; **P < 0.01, ***P < 0.001; two-tailed t-test). **(C)** The cell proliferation rate was analyzed by CCK-8 assay. All value were mean ± SD (n = 3; ***P < 0.001; two-tailed t-test). **(D)**, **(E)** Colony formation assays were performed (n = 3; ***P < 0.001; two-tailed t-test). **(F, G)** Distribution of cells in three cell cycle phases was examined by flow cytometry assay, and the graph shows quantification for each phase. **(H)** For measurement of apoptotic cells, cells were stained with both AV and PI and analyzed by an image flow assay. **(I)** Graph illustrating the quantification of apoptotic cells (n = 3; ***P < 0.001; two-tailed t-test). Abbreviations: AV, Annexin V FITC; CCK-8, cell counting kit-8, PI, propidium iodide; NC, negative control.

To identify the expression level of MAD2L1 in CRC, we performed qRT-PCR to confirm the expression of MAD2L1 in 20 paired clinical samples, in which the mean expression level of MAD2L1 was notably higher in CRC tissues than in normal tissues (**Figure 6A**). Next, we measured the expression of MAD2L1 in various cell lines, including the normal cell line NCM460 and the CRC cell lines HT-29, HCT116, SW620, and SW480. The expression of MAD2L1 was higher in tumor cells than in normal cells (**Figure 6B**), which is similar to the results from the four datasets in GEO and the GEPIA results, suggesting that our results for these genes are reliable.

### Knockdown of MAD2L1 Suppressed Cell Growth by Impairing Cell Cycle Progression and Inducing Cell Apoptosis

To determine whether MAD2L1 could be a therapeutic target in CRC, we inactivated MAD2L1 by using siRNAs in SW620 cells. We found that the MAD2L1 knockdown, compared to the control knockdown, significantly inhibited cell proliferation (**Figure 6C**) and reduced cell numbers of SW620 cells (**Figures 6D, E**), which indicated that MAD2L1 might promote cell proliferation. To examine how MAD2L1affects cell growth, the cell cycle phase distribution and apoptosis were analyzed by flow cytometric analysis. Knockdown of MAD2L1 resulted in a decrease in the percentage of cells in the G1 and G2 phases and an increase in the percentage of cells in the S phase (**Figures 6F, G**), which indicated that MAD2L1 knockdown prevented cell passage from the S phase into the G2 phase. Therefore, MAD2L1 was shown to promote S/G2 phase transition. The apoptosis assay results indicated that the apoptotic cells significantly increased in SW480 cells with si-MAD2L1 transfection (**Figures 6H, I**). These data indicate that MAD2L1 knockdown could impair cell cycle progression and induce cell apoptosis.

## Discussion

Even with a gradual decline in the past few years, CRC remains the fourth leading cause of cancer-related death worldwide (Marmol et al., 2017). The occurrence and development of CRC is a dynamic process. At different stages of CRC, the expression levels of some molecules are different. (Moroishi et al., 2015) In this case, early screening and diagnosis are becoming increasingly difficult. Therefore, it is necessary to find accurate and meaningful CRC biomarkers. Our study systematically focused on expression profiles obtained from microarray studies of CRC. Our analysis included 74 CRC samples and 65 normal samples from the GSE117606 dataset of the GEO database. A total of 683 DEGs were identified, including 420 upregulated genes and 263 downregulated genes. To better explore these DEGs, we carried out GO function and KEGG pathway analysis of these DEGs.

GO analysis showed that the upregulated DEGs were particularly enriched in mitotic collagen catabolic process, extracellular matrix organization, proteinaceous extracellular matrix, extracellular region, extracellular matrix structural constituent, and extracellular matrix binding, while the downregulated DEGs were involved in bicarbonate transport, muscle contraction, extracellular exosome, extracellular space, chloride channel activity, and carbonate dehydratase activity. In addition, the KEGG pathways for the upregulated DEGs included the cell cycle, ECM-receptor interaction, and focal adhesion, while the pathways of the downregulated DEGs were mainly in mineral absorption, proximal tubule bicarbonate reclamation, and retinol metabolism.

A PPI is defined as the process by which two or more kinds of protein molecules form a protein complex by noncovalent bonding. PPI networks could provide a visible framework for a better understanding of the functional organization of the proteome (Liu et al., 2009). The enriched pathways of the top 3 modules showed that CRC was associated with the cell cycle-related pathway and the p53 signalling pathway.

Cell cycle-related genes that promote the proliferation of endothelial cells contribute to the progression of tumor growth and metastasis of CRC (Hong et al., 2009). *CDK1* encodes a serine/threonine kinase that controls the eukaryotic cell cycle by regulating mitotic onset, as well as the centrosome cycle (Santamaria et al., 2007). *CDK1* promotes cell proliferation *via* the phosphorylation and inhibition of the forkhead box O1 transcription factor (Liu et al., 2008). The alteration of CDK1 has been found in numerous cancer types, including breast cancer (Kim et al., 2008), esophageal adenocarcinoma (Hansel et al., 2005), hepatocellular carcinoma (Wu et al., 2019), pancreatic ductal adenocarcinoma (Piao et al., 2019), and oral squamous cell carcinoma (Chang et al., 2005). Iacopetta et al. revealed that p53 mutations that lose transactivation ability are more common in advanced CRC and associated with poor survival (Iacopetta et al., 2006). Slattery ML et al. suggested that the activation of p53 from cellular stress could target downstream genes that could in turn influence cell cycle arrest, apoptosis, and angiogenesis through mRNA:miRNA interactions (Slattery et al., 2018). In the p53 signaling pathway, the RRM2 gene was an oncogene that was overexpressed in colorectal cancer, with its elevated expression correlated with the invasion depth, poorly differentiated type, and tumor node metastasis stage (Lu et al., 2012).

Twenty DEGs with high connectivity were selected as hub genes for PPI network analysis. By analyzing the correlations and expression levels in GEPIA, we determined that the hub genes were obviously positively correlated and significantly overexpressed in CRC samples.

We searched the literature in PubMed for associations among the twenty hub genes in CRC. In Yanqi Gan et al.'s study, they revealed that expression of CCNA2 in CRC tissues is higher than that in normal tissues and that CCNA2 knockdown could significantly suppress CRC cell growth by impairing cell cycle progression and inducing cell apoptosis (Gan et al., 2018). *TOP2A* is a gene that involves copy number variations and chromosomal instability in many cancers (Simon et al., 2002; Bofin et al., 2003; Chen et al., 2015; Sonderstrup et al., 2015). In colorectal cancer, the protein expression level of *TOP2A* was related to aggressive tumor phenotypes and advanced tumor stages (Coss et al., 2009). In our research, we found that *TOP2A* expression was upregulated in colorectal cancer. The expression of PLK1 was correlated with tumor size, lymph node metastasis, depth of invasion, and TNM stage, consistent with the results from Takahashi et al. (Takahashi et al., 2003). Ding-pei Han et al.'s study revealed that PLK1 has additional functions and is involved in the proliferation, migration and invasion of colorectal cancer cells (Han et al., 2012). The spindle proteins AURKA, BUB1, and MAD2L1 are important components of the spindle assembly checkpoint (Xue et al., 2016), which has been frequently established as an important mechanism that drives aneuploidy and carcinogenesis in CRC (Chen et al., 1998; Burum-Auensen et al., 2007). Anke H, Sillars-Hardebol et al.'s study revealed TPX2 and AURKA as major players in this critical step in colorectal carcinogenesis (Sillars-Hardebol et al., 2012). RRM2 overexpression was significantly associated with invasion depth and differentiation, and clinical tissue specimens also showed that the expression levels of RRM2 may be associated with tumor stage, which was shown in Ai-Guo Lu et al.'s study (Lu et al., 2012). KIF11 is a mitotic kinesin and is required for the separation of duplicated centrosomes during spindle formation (Zhu et al., 2005). Imai T et al.'s results verified that knockdown of *KIF11* by siRNA inhibits sphere formation, indicating that *KIF11* is important in the activity of esophageal cancer and CRC (Imai et al., 2017). MELK was overexpressed and highly phosphorylated in colorectal adenocarcinomas, and its expression was significantly correlated with tumor stage and lymph node metastasis (Gong et al., 2018). NUSAP1 is a microtubule-binding protein that plays a vital role in the assembly of mitotic spindle (Song and Rape, 2010). NUSAP1 gene silencing induced cell apoptosis and inhibited cell proliferation, cell migration, cell invasion, and EMT in colorectal cancer by inhibiting DNMT1 gene expression (Han et al., 2018). Human replication factor C (RFC) is a multimeric protein consisting of five distinct subunits that are highly conserved through evolution (Yao and O'Donnell, 2012). Jun Xiang et al.'s results revealed that the overexpression of *RFC4* commonly occurs in CRC and that a high expression level of RFC4 is associated with poor differentiation and late TNM stages in patients with CRC. Higher levels of RFC4 protein expression correlate with a worse overall survival in CRC (Xiang et al., 2014). Human pituitary tumor transforming gene-1 (PTTG1) is a novel oncogene. Ren Q et al.'s study preliminarily explored the effects of PTTG1 in colorectal cancer cell proliferation and metastasis and found that the downregulation of PTTG1 expression suppressed colorectal cancer cell proliferation, migration and invasion (Ren and Jin, 2017). Gali-Muhtasib H et al.'s study confirmed the *in vivo* existence of the CHEK1/p53 link in human colorectal cancer, showing that tumors lacking p53 had higher levels of CHEK1, which was accompanied by poorer apoptosis. CHEK1 overexpression was correlated with advanced tumor stages, proximal tumor localization, and worse prognosis (Gali-Muhtasib et al., 2008). Overexpression of *CEP55* activates p21 and enhances the cell cycle transition. In contrast, the knockdown of *CEP55* inhibits cell growth in gastric (Tao et al., 2014) and breast cancer (Wang et al., 2016). DTL is located at chromosomal region 1q32.1–32.2 and encodes a putative 730-amino-acid nuclear protein that contains six highly conserved WD40-repeat domains (Ueki et al., 2008). It has been reported that DTL plays an essential role in cell proliferation, cell cycle arrest and metastatic potential in hepatocellular carcinoma, breast cancer, gastric cancer and rhabdomyosarcoma (Pan et al., 2006; Ueki et al., 2008; Li et al., 2009; Missiaglia et al., 2009; Song et al., 2010). Baraniskin A et al.'s data identified miR-30a-5p as a tumor-suppressing miRNA in colon cancer cells, exerting its function *via* the modulation of DTL expression, which is frequently overexpressed in CRC (Baraniskin et al., 2012).

MAD2L1 is highly expressed in colon cancer according to biological information. Moreover, MAD2L1 has a high positive correlation, with a Pearson correlation coefficient of 0.88. Through bioinformatics analysis of GSE117606, we know that MAD2L1 is one of the 20 core genes, and that MAD2L1 plays a role in the occurrence and development of colon cancer by participating in the cell cycle pathway. In examining the expression level of MAD2L1, we found that MAD2L1 has a higher expression in the CRC clinical samples and cell lines. Afterward, by searching PubMed, we found that there were no relevant studies reporting that MAD2L1 is involved in the cell cycle pathway, so we chose MAD2L1 for the next cell experiments. We further confirmed that knockdown of MAD2L1 could significantly suppress CRC cell growth by impairing cell cycle progression and inducing cell apoptosis. MAD2L1 has the potential to be a new biomarker for diagnosis and therapy in CRC.

There is a limitation of this study that needs to be considered: the analysis of a single dataset from GEO will result in partial bias, and too few samples will not lead to new findings. However, the data set we selected contains a large number of samples, so this limitation can be compensated to a certain extent.

In summary, using the GSE117606 profile data set and multiple bioinformatics analyses, our present work identified twenty hub genes as DEGs. These DEGs are significantly enriched in several pathways that are mainly associated with the cell cycle, ECM-receptor interaction, and mineral absorption pathways in CRC, and they might play key roles in the development and progression of CRC. MAD2L1 shows higher expression levels in CRC, is involved in colon cancer cell growth and cell cycle progression, and could be used as a new biomarker since it has a significant meaning for clinical treatment.

## Conclusion

In this study, using a GSE data set and multiple bioinformatics analyses, we identified twenty hub genes that were significantly enriched in the cell cycle, ECM–receptor interaction, and mineral absorption pathways in CRC. Moreover, the expression level of MAD2L1 was significantly increased in CRC, and knockdown of MAD2L1 suppressed colon cancer cell growth by impairing cell cycle and apoptosis progression. Our findings also establish that MAD2L1 could be a new biomarker for CRC diagnosis and guide combination therapy for CRC.

## Data Availability Statement

Publicly available datasets were analyzed in this study. This data can be found at Gene Expression Omnibus: https://www.ncbi.nlm.nih.gov/geo/query/acc.cgi?acc=GSE117606).

## Ethics Statement

The studies involving human participants were reviewed and approved by Ethics committee of Renmin Hospital of Wuhan University Renmin Hospital of Wuhan University. The patients/participants provided their written informed consent to participate in this study.

## Author Contributions

XD is responsible for the design of experiments, bioinformatic analysis, collection of samples and specific experimental operations. HD is responsible for data collation and statistical analysis. HL is responsible for providing experimental funds and technical guidance.

## Conflict of Interest

The authors declare that the research was conducted in the absence of any commercial or financial relationships that could be construed as a potential conflict of interest.
